# The association between patellofemoral grind and synovitis in knee osteoarthritis: data from the osteoarthritis initiative

**DOI:** 10.3389/fmed.2023.1231398

**Published:** 2023-08-29

**Authors:** Hui Deng, Yongzhong Wu, Zaiwei Fan, Wubing Tang, Jun Tao

**Affiliations:** ^1^Department of Orthopedics, Second Affiliated Hospital of Nanchang University, Nanchang, China; ^2^Second People's Hospital of Jingdezhen, Jingdezhen, China; ^3^Third Hospital of Nanchang, Nanchang, China

**Keywords:** patellofemoral grind, osteoarthritis, clinical physical examination, synovitis, gender difference

## Abstract

**Objective:**

Patellofemoral grind refers to the tender behind the knee cap while contracting the quadriceps muscle during the patellar grind test. The present investigation aims to elucidate the association between patellofemoral grind and synovitis in the knee osteoarthritis (KOA).

**Method:**

A total of 1,119 knees with complete patellofemoral grind and synovitis assessment records from the Osteoarthritis Initiative (OAI) were investigated in this study. The Magnetic Resonance Imaging at baseline, 12 months, and 24 months of follow-up were employed to evaluate synovitis. Frequent patellofemoral grind was operationally defined as occurring more than twice at three different time points. In addition, a sensitivity stratification was conducted to examine gender differences.

**Results:**

The study participants had an average age of 61 years, with 62.4% being female. The findings revealed that baseline patellofemoral grind was significantly associated with changes in synovitis at follow-up (odds ratio [OR]: 1.44, confidence interval [CI]: 1.04–1.98) and was also linked to synovitis worsening over 24 months (OR: 1.67, CI: 1.13–2.46) in all subjects. For the subjects with frequent patellofemoral grind, this correlation was more significant (OR: 1.50, CI: 1.03–2.16; OR: 1.71, CI: 1.09–2.67). In the context of sensitivity stratification, it was observed that the baseline and frequent patellofemoral grind in females exhibited a significant correlation with synovitis. However, no significant correlation was found in males.

**Conclusion:**

Patellofemoral grind may serve as a potential risk factor of synovitis in knee osteoarthritis, particularly among female patients, and thus, necessitates close monitoring and management by clinical physicians.

## Introduction

Osteoarthritis (OA) is the most common type of arthritis. Approximately 300 million people have OA worldwide, and its incidence increases annually, leading to pain and functional disability and affecting daily life (1, 2). The main characteristic changes of OA are joint cartilage degeneration, osteophyte hyperplasia, and synovial inflammation, resulting in pain, joint dysfunction, and deformity (3). Despite the high prevalence of OA, there is currently a lack of effective pharmacological interventions that can modify the structural damage and symptom progression of knee joints in affected patients (4).

The etiology of osteoarthritis was very complex and involves multiple risk factors. Prior research has established a link between patellofemoral grind and prolonged knee cartilage loss as well as total knee arthroplasty (5). Patellofemoral grind refers to the tender behind the knee cap when the quadriceps muscle is contracted during the patellar grind test. This standardized patellofemoral grind test has a good correlation with the physical examination of knee osteoarthritis (KOA) (6). Generally, a positive patellofemoral grind test indicates the presence of patellofemoral pathology, such as patellar chondromalacia, patellofemoral pain syndrome, and potentially patellofemoral arthritis (7–9). While patellofemoral arthritis often co-occurs with tibiofemoral arthritis, the longitudinal association between patellofemoral grinding and osteoarthritis of the tibiofemoral joint remains unexplained (10). Therefore, further research is necessary to elucidate the relationship between patellofemoral grinding and the inflammatory phenotype of knee osteoarthritis, thereby enhancing the treatment and management of affected patients.

Numerous phenotypes of knee osteoarthritis (KOA) have been identified, such as those driven by trauma, cartilage, senescence, and synovitis (11). Notably, the interaction between macrophages and chondrocytes induced by synovitis has been acknowledged as a significant contributor to KOA in recent years (12). Given the diversity among these phenotypes, it is imperative to accurately identify the pertinent risk factors associated with each phenotype to facilitate personalized treatment and management. Patellofemoral grind, a form of anterior knee pain, has traditionally been linked to structural alterations in the knee joint. Nevertheless, recent research has emphasized the connection between knee pain and inflammation (13). Despite this, the relationship between patellofemoral grind and the KOA synovitis phenotype has yet to be fully established. As such, exploring the potential correlation between patellofemoral grind and synovitis in KOA could contribute to our comprehension of this condition.

The objective of this investigation was to examine the plausible correlation between patellofemoral grind and synovitis. It was hypothesized that a longitudinal connection existed between patellofemoral grind or frequent patellofemoral grind (occurring more than twice at three time points) at baseline and alterations in knee synovitis, and that this association could also be observed in a cross-sectional study. Furthermore, a sensitivity analysis was performed, and assuming there is a gender difference in this association.

## Methods

### Database and participants

The Osteoarthritis Initiative (OAI) is a multicenter observational cohort study focused on knee osteoarthritis (KOA). The study enrolled 4,796 participants, both men and women aged 45–79 years, who exhibited symptomatic KOA or were at risk of developing it, from four clinical recruitment sites.^1^ The exclusion criteria for the study included inflammatory arthritis, severe stenosis of bilateral knee joint space, unilateral knee joint replacement, and the requirement of walking aids. The institutional review committee at the respective locations approved the OAI study, and all participants provided informed consent by signing a form. Additional information regarding the study protocol can be accessed at https://nda.nih.gov/oai/study_documentation.html.

**Figure 1:**
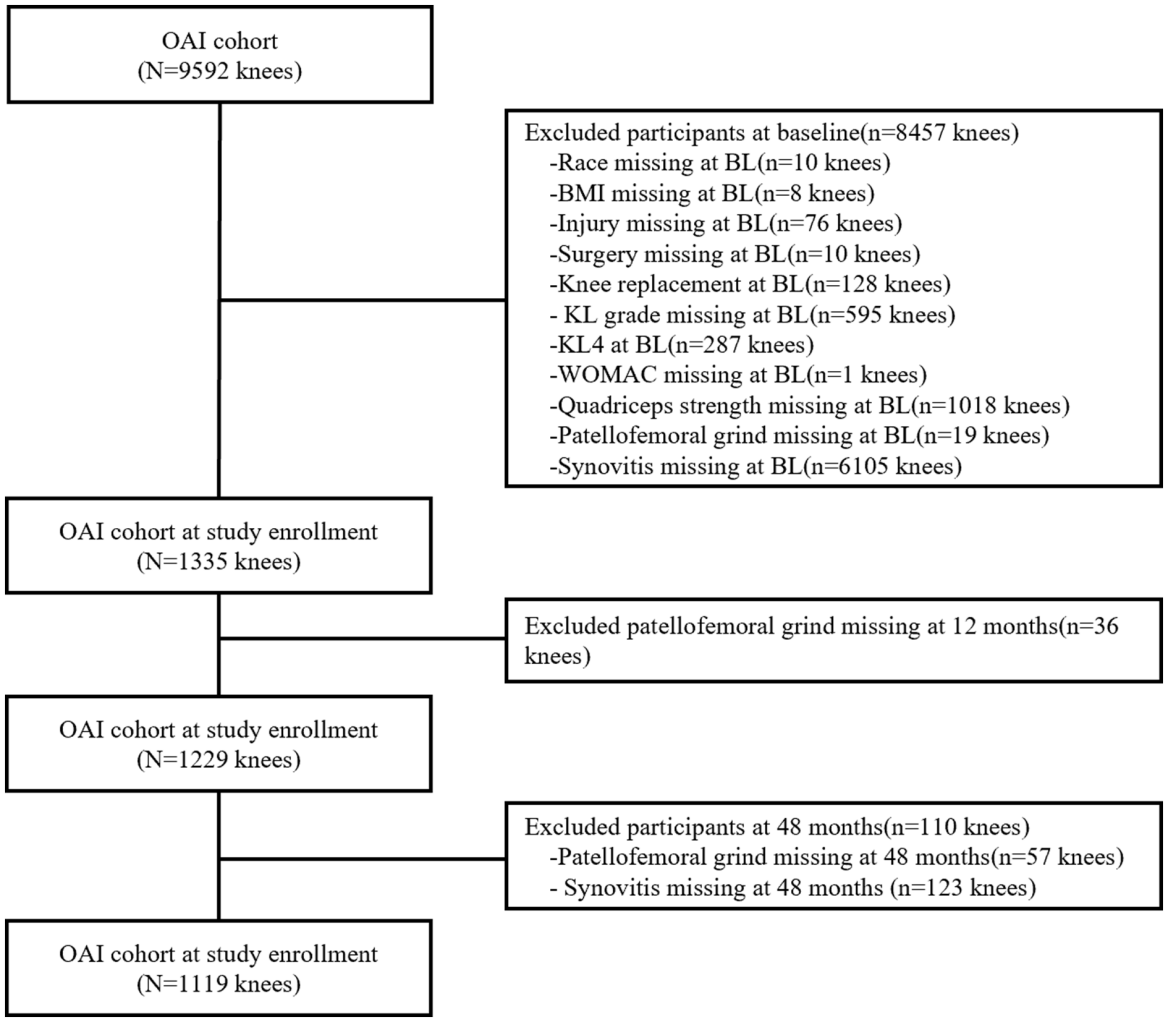
Flow chart of selection criteria.

Participants who lacked data on race, body mass index (BMI), knee injury and surgery history, Kellgren and Lawrence (KL) grade, Western Ontario and McMaster Universities Osteoarthritis Index (WOMAC) score (0–20), quadriceps strength, patellofemoral grind, and synovitis score were excluded. In addition, subject knees with KL4 or had undergone knee replacement surgery were excluded. Annual follow-ups were conducted on the subjects, however, only MRI data from 24 months of follow-up were deemed relatively complete. Ultimately, a total of 1,119 knees with complete records were utilized to examine the correlation between patellofemoral grind and synovitis (Figure 1). It is noteworthy that the excluded subjects shared similar demographic characteristics with those who were selected for the study.

In our assessment, we examined various potential sources of bias, encompassing significant demographic variables such as gender, age, race, and BMI, as well as the history of knee injury and surgery at the time of enrollment. Specifically, knee injury history was defined as the extent to which a participant’s knee was severely injured, thereby restricting their walking ability for a minimum of 1 week. Additionally, we accounted for the variance in knee osteoarthritis severity between imaging and symptomatology by incorporating baseline KL grade and WOMAC scores as potential sources of bias. In addition, as Gong Z et al. have recently reported a negative correlation between synovitis and quadriceps strength, we incorporated quadriceps strength as an additional covariate (14). Trained personnel assessed quadriceps strength using the Good Strength Chair (Metitur Oy, Jyvaskyla, Finland) with high retest reliability (*r* = 0.88–0.92) (15). Following two warm-up tests, the highest score from three repeated maximum effort tests of knee quadriceps strength was utilized for analysis, and torque was calculated by dividing the length of the lever arm by body weight in Newton meters [Nm/kg].

### Clinical assessment of patellofemoral grind

The medical personnel underwent rigorous centralized training, after which they conducted knee examinations with the guidance of a physician at each site. Participants were instructed to assume a supine position on the examination table and relax their quadriceps muscles. The examiner placed their hand over the patella, mimicking the technique used during the crepitus examination, and applied gentle pressure towards the table until the patella reached its limit of motion. While maintaining pressure on the patella, participants were instructed to contract their quadriceps muscles against the resistance of patellar motion. During the study, participants were instructed to contract or tighten their muscles and report any pain experienced, which was subsequently recorded on the data collection form. The comprehensive examination protocol was accessible online via https://nda.nih.gov/oai/study_documentation.html. The OAI study collected participant data at three distinct intervals, spanning from baseline to 24 months follow-up. Frequent patellofemoral grind was defined as occurring more than twice during these time points. Prior research has established that joint and periarticular tenderness or pain, as evaluated through standardisation, is a reliable measure of knee function (6).

### Assessment of synovitis

The MRI acquisition in four clinical sites utilized the 3 Tesla magnetic resonance imaging (MRI) system (Trio; Siemens Healthcare). Hoffa synovitis and effusion synovitis were surrogate markers for identifying synovial inflammation on non-contrast-enhanced MRI (16). Hoffa synovitis was identified by axial fat suppression (FS) turbine rotating echo (TSE) high signal on the sagittal plane and coronal plane, while effusion synovitis was identified by axial multiplanar recombination (MPR) in the three-dimensional dual echo steady water excitation (3D-DESSWE) sequence following intravenous injection of a contrast agent. Two musculoskeletal radiologists, AG and FR, with 15 and 13 years of experience, respectively, performed the MRI Osteoarthritis Knee Score (MOAKS) system in a blinded manner to relevant clinical features. The synovitis measurements demonstrated high consistency, with 95% (95% confidence interval [CI]: 0.61–1.00) and 68% (95% CI, 0.38–0.99) intra-observer consistency for effusion and Hoffa synovitis, respectively (17). The synovitis summary score, which ranged from 0 to 6, was calculated as the sum of Hoffa synovitis scores and effusion scores (18). The worsening of synovitis was operationally defined as a minimum increase of one score in the synovitis summary score from baseline to the 24 months follow-up. All images can be downloaded from https://nda.nih.gov/oai/accessing_images.html

### Statistical analysis

Descriptive statistics were utilized to express demographic variables as either the mean and standard deviation (SD) for continuous variables or as a percentage for categorical variables. Following the successful completion of the collinearity diagnosis and parallel line test, the ordered logistic regression was employed to investigate the cross-sectional and longitudinal relationships between patellofemoral grind and synovitis. The binary logistic regression was utilized to evaluate the connections between synovitis worsening and patellofemoral grind. Model 1 was unadjusted for any confounding variables, while Model 2 was adjusted for baseline characteristics such as gender, age, race, BMI, knee injury history, knee surgery history, and WOMAC score. Model 3 further adjusted for baseline quadriceps strength. In order to analyze gender differences, a sensitivity analysis was conducted with gender stratification. Statistical significance was determined by *p*-values of <0.05 and 95% confidence intervals (CIs) of >0. The Student’s *t*-test was employed to assess the variations in synovitis scores among subject knees with and without patellofemoral grind on an annual basis. All statistical analyses were conducted using the SPASS software (version 25, SPSS Science, Chicago, Illinois) in accordance with academic standards.

## Result

### Participant characteristics

Table 1 presents the amalgamated data on demographic characteristics, patellofemoral grind changes, and study variables. The subjects were aged between 45 and 79 years, with 62.4% being female and 84.3% being of white ethnicity. The mean (SD) age, BMI, quadriceps strength, and WOMAC scores of the subjects at baseline were 61.5 ± 8.72 years, 29.5 ± 4.68 kg/m2, 1.2 ± 0.46 Nm/kg, and 2.4 ± 3.06, respectively. Of the total subjects, 45.5% had a history of knee injury, while 26% had a history of knee surgery. The majority of the subject knees had KL grades of 1 (30.2%), 2 (29.2%), and 3 (24.0%). At baseline, a total of 161 knees of the subjects exhibited patellofemoral grind, whereas 854 knees displayed synovitis. Furthermore, a total of 32 knees of the subjects manifested patellofemoral grind without synovitis, while 724 knees presented synovitis without patellofemoral grind. The summary score for synovitis escalated by 0.20 over a period of 24 months, and 298 knees of the subjects experienced a worsening in synovitis.

**Table 1:** The demographic characteristics of the study.

	*N* = 1,119
Age, mean (SD)	61.5 (8.72)
Female, *n* (%)	698 (62.4%)
Male, *n* (%)	421 (37.6%)
Race, *n* (%)	
White	943 (84.3%)
Others	176 (15.7%)
BMI (kg/m^2^), mean (SD)	29.5 (4.68)
Injury, *n* (%)	510 (45.5%)
Surgery, *n* (%)	292 (26.0%)
Quadriceps strength, mean (SD)	1.25 (0.46)
WOMAC, median (IQR)	1 (0, 4)
Baseline Kellgren and Lawrence (K-L) grade, *n* (%)	
0	186 (16.6%)
1	338 (30.2%)
2	327 (29.2%)
3	269 (24.0%)
Baseline patellofemoral grind, *n* (%)	161 (14.3%)
Baseline synovitis summary score, median (IQR)	
(Range 0–6)	1 (1,2)
Frequent patellofemoral grind, *n* (%)	113 (10.0%)
Synovitis worsening, *n* (%)	298 (26.6%)

BMI, body mass index; SD, standard deviation; IQR, interquartile range; WOMAC, Western Ontario and McMaster Universities Osteoarthritis Index.

### The association between patellofemoral grind and synovitis over 24 months

Table 2 delineates the correlation between the presence of patellofemoral grind at baseline or frequent intervals and alterations in synovitis over 24 months in the whole population or gender stratification. After adjusting for baseline sex, age, race, body mass index, WOMAC scores, injury, surgery, and KL grades, Model 2 revealed that baseline patellofemoral grind was significantly associated with changes in synovitis over 24 months (odds ratio [OR]: 1.42, 95% confidence interval [CI]: 1.03–1.95). Upon further adjustment for quadriceps strength in Model 3, this longitudinal correlation persisted (OR: 1.44, 95% CI: 1.04–1.98). Additionally, both Model 2 (OR: 1.49, 95% CI: 1.03–2.16) and Model 3 (OR: 1.50, 95% CI: 1.03–2.16) demonstrated a significant correlation between frequent patellofemoral grind and changes in synovitis over 24 months. Notably, the odds ratio for these associations was higher in the female population.

**Table 2:** Association of patellofemoral grind with synovitis over 24 months.

	Model 1^a^		Model 2^b^		Model 3^c^	
	OR(95% CI)	*p*	OR(95% CI)	*p*	OR(95% CI)	*p*
Baseline patellofemoral grind						
All	1.828 (1.356–2.464)	**<0.001**	1.421 (1.033–1.954)	**0.031**	1.443 (1.049–1.984)	**0.024**
Men	1.098 (0.662–1.821)	0.717	0.828 (0.484–1.419)	0.493	0.835 (0.488–1.430)	0.512
Women	2.539 (1.748–3.690)	**<0.001**	2.032 (1.363–3.031)	**<0.001**	2.081 (1.394–3.105)	**<0.001**
Frequent patellofemoral grind						
All	1.843 (1.302–2.608)	**0.001**	1.498 (1.036–2.166)	**0.032**	1.500 (1.037–2.168)	**0.031**
Men	1.323 (0.720–2.430)	0.367	1.058 (0.556–2.009)	0.865	1.046 (0.550–1.989)	0.890
Women	2.225 (1.454–3.403)	**<0.001**	1.742 (1.108–2.739)	**0.016**	1.758 (1.118–2.764)	**0.015**

a Model 1: unadjusted.

b Model 2: adjusted for sex, age, race, body mass index, Western Ontario and McMaster Universities Osteoarthritis Index (WOMAC), injury, surgery, and Kellgren-Lawrence (KL) grade at baseline.

c Model 3: Model 2 + baseline quadriceps strength. The bold *p*-value indicates that the result is statistically significant.

### Association between patellofemoral grind and synovitis worsening over 24 months

Table 3 presents the correlation between the occurrence of patellofemoral grind at baseline or frequently and the exacerbation of synovitis from baseline to 24 months. In Model 2, the presence of patellofemoral grind at baseline (OR: 1.66, 95% CI: 1.12–2.45) or frequent patellofemoral grind (OR: 1.70, 95% CI: 1.09–2.67) was identified as a significant risk factor for the worsening of synovitis. After additional adjustments to the quadriceps strength, Model 3 still indicated that baseline patellofemoral grind (OR: 1.67, 95% CI: 1.13–2.46) or frequent patellofemoral grind (OR: 1.71, 95% CI: 1.09–2.67) were risk factors for synovitis worsening. In Model 3, women exhibited a greater odds ratio for the correlation between baseline (OR: 2.47, 95% CI: 1.52–3.99) or frequent patellofemoral grind (OR: 2.30, 95% CI: 1.34–3.96) and synovitis worsening, as compared to the entire population.

**Table 3:** Association of patellofemoral grind with synovitis worsening over 24 months.

	Model 1^a^		Model 2^b^		Model 3^c^	
	OR(95% CI)	*p*	OR(95% CI)	*p*	OR(95% CI)	*p*
Baseline patellofemoral grind						
All	1.477 (1.032–2.112)	**0.033**	1.663 (1.126–2.455)	**0.011**	1.670 (1.130–2.466)	**0.010**
Men	1.329 (0.673–2.623)	0.413	1.267 (0.613–2.616)	0.523	1.270 (0.615–2.625)	0.518
Women	2.203 (1.313–3.116)	**0.001**	2.438 (1.507–3.943)	**<0.001**	2.470 (1.526–3.999)	**<0.001**
Frequent patellofemoral grind						
All	1.520 (1.006–2.297)	**0.047**	1.709 (1.091–2.678)	**0.019**	1.710 (1.091–2.679)	**0.019**
Men	0.782 (0.345–1.772)	0.556	0.878 (0.368–2.094)	0.770	0.874 (0.366–2.085)	0.761
Women	2.009 (1.230–3.280)	**0.005**	2.295 (1.336–3.945)	**0.003**	2.306 (1.342–3.963)	**0.002**

a Model 1: unadjusted.

b Model 2: adjusted for sex, age, race, body mass index, Western Ontario and McMaster Universities Osteoarthritis Index (WOMAC), injury, surgery, and Kellgren-Lawrence (KL) grade at baseline.

c Model 3: Model 2 + baseline quadriceps strength. The bold *p*-value indicates that the result is statistically significant.

### The horizontal association between patellofemoral grind and synovitis

**Figure 2:**
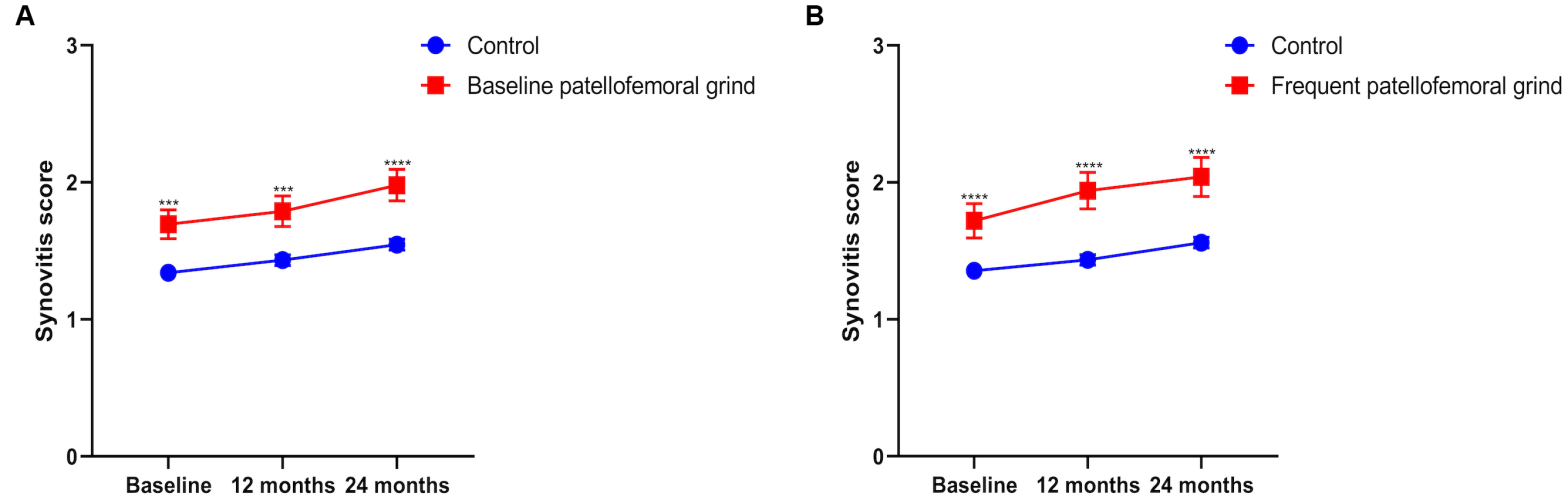
The summary score of synovitis from baseline to 24 months. **(A)** Annual synovitis score with or without patellofemoral grind at baseline. **(B)** Annual synovitis score for frequent patellofemoral grind.

The annual synovitis score was higher in the population with patellofemoral grind at baseline than in the subjects without patellofemoral grind at baseline (Figure 2A). The same results were observed for the population with frequent patellofemoral grinds (Figure 2B). Table 4 displays the horizontal correlation between patellofemoral grind and synovitis changes at baseline or 24 months. Multivariate analysis across all populations revealed no significant correlation between baseline patellofemoral grind and synovitis changes. However, at 24 months, both Model 2 (OR: 1.96, 95% CI: 1.45–2.65) and Model 3 (OR: 1.95, 95% CI: 1.44–2.64) demonstrated a significant correlation between patellofemoral grind and synovitis changes. Interestingly, among women, we observed a correlation between baseline patellofemoral grind and synovitis changes, and this horizontal association also existed at 24 months.

**Table 4:** Association of patellofemoral grind and synovitis.

	Model 1^a^		Model 2^b^		Model 3^c^	
	OR (95% CI)	*p*	OR (95% CI)	*p*	OR (95% CI)	*p*
Baseline						
All	1.892 (1.401–2.557)	**<0.001**	1.328 (0.964–1.831)	0.083	1.341 (0.973–1.849)	0.073
Men	–	–	1.038 (0.604–1.782)	0.893	1.040 (0.606–1.787)	0.886
Women	2.206 (1.515–3.209)	**<0.001**	1.511 (1.011–2.259)	**0.044**	1.534 (1.025–2.293)	**0.037**
Over 24 months						
All	2.217 (1.656–2.967)	**<0.001**	1.965 (1.455–2.654)	**<0.001**	1.957 (1.449–2.643)	**<0.001**
Men	1.753 (1.111–2.768)	**0.016**	1.594 (1.001–2.540)	**0.049**	1.562 (0.981–2.489)	0.060
Women	2.567 (1.756–3.751)	**<0.001**	2.284 (1.540–3.391)	**<0.001**	2.305 (1.553–3.421)	**<0.001**

a Model 1: unadjusted.

b Model 2: adjusted for sex, age, race, body mass index, Western Ontario and McMaster Universities Osteoarthritis Index (WOMAC), injury, surgery, and Kellgren-Lawrence (KL) grade at baseline.

c Model 3: Model 2 + baseline quadriceps strength. The bold *p*-value indicates that the result is statistically significant.

## Discussion

To date, there is a dearth of research investigating the cross-sectional and longitudinal correlation between patellofemoral grind and synovitis in individuals with knee osteoarthritis (KOA). Patellofemoral grind is frequently utilized as a prognostic indicator for chondromalacia patellae or patellofemoral pain syndrome (19). Nevertheless, the diagnostic accuracy of the patellofemoral grind test for detecting chondromalacia patellae and patellofemoral pain syndrome is low, with limited clinical relevance (7). The current investigation aimed to examine the correlation between the occurrence or frequency of patellofemoral grind and alterations in synovitis. To account for potential confounding variables, we included established risk factors for knee osteoarthritis, such as age, race, sex, body mass index, KL grade, knee injury history, surgery history, and WOMAC score. Furthermore, we incorporated quadriceps muscle strength as an additional adjustment parameter based on prior literature to mitigate potential observational bias. The findings of our multivariate analysis indicate a cross-sectional or longitudinal correlation between the presence of patellofemoral grind and changes in synovitis. Furthermore, our results suggest that the baseline or frequent patellofemoral grind may be linked to the worsening of synovitis, particularly in female patients. These results underscore the importance of patellofemoral grind as a clinical examination indicator, which extends beyond its conventional use in assessing the pathological state of the patellofemoral joint.

Prior research has established a correlation between patellofemoral grind and the risk of cartilage injury and total knee arthroplasty in tibiofemoral arthritis (5). However, the influence of patellofemoral grind on the longitudinal of tibiofemoral joint osteoarthritis was difficult to explain. Increasing evidence suggests that the inflammatory phenotype serves as a mediator in the pathogenesis of knee osteoarthritis, and inflammation appears to be a crucial factor in the onset and progression of joint diseases (20). The synovial inflammatory response observed in osteoarthritis is characterized by synovial hyperplasia, synovial sac thickening, and infiltration of various immune cells (21). The fragmentation of cartilage triggers the polarization of macrophages in the synovial membrane towards M1, leading to the secretion of pro-inflammatory mediators by activated macrophages. This, in turn, exacerbates the degradation of chondrocytes and matrix, thereby establishing a vicious cycle of cartilage degradation and inflammation (12). The occurrence of cartilage damage in the patellofemoral joint has the potential to initiate or exacerbate synovial inflammation, thereby contributing to the advancement of knee osteoarthritis. Notably, patellofemoral arthritis and tibiofemoral arthritis frequently manifest concurrently (22). A recent research has indicated that persistence of synovitis are linked to the abnormal cartilage structures in the patellofemoral and tibiofemoral joints (23). While the patellofemoral grind test may not differentiate between patellofemoral arthritis and tibiofemoral arthritis (24), our findings suggest a cross-sectional and longitudinal association between patellofemoral grind and the progression and changes of synovitis. Hence, it is probable that individuals with patellofemoral grind may experience advancement in patellofemoral and tibiofemoral osteoarthritis as a result of the persistence or worsening of the synovitis phenotype associated with osteoarthritis.

To investigate the correlation between patellofemoral grind and synovitis, we set baseline and frequent patellofemoral grind as crucial exposure points, which are the most readily accessible indicators of the patellofemoral joint in clinical assessments. The primary outcome was determined by the sum score of effusion synovitis and Hoffa synovitis in the analysis. The patellofemoral grind typically results from improper patellar tracking or positioning, while the trajectory of the patella being closely linked to the strength of the quadriceps muscle (25). Therefore, during the analysis, we incorporated quadriceps strength as an additional covariate in model 3. Our study first provides evidence that patients with a positive patellofemoral grind test are at risk of experiencing exacerbated synovitis. The key force of this finding is attributed to the utilization of semiquantitative measurements of synovitis obtained from MRI images, which are highly responsive to changes in synovitis, despite the potential for measurement errors. Felson et al. conducted a comparative analysis of 239 cases and 731 control knees within the MOST cohort, revealing that cartilage lesions, meniscus injury, synovitis, and bone marrow lesion (BML) were all identified as risk factors for osteoarthritis (26). Similarly, Atukorala et al. conducted a nested case–control analysis on 133 knee joints with and without knee osteoarthritis, and discovered that effusion synovitis and Hoffa synovitis within the first year of diagnosis were significantly associated with subsequent osteoarthritis development (27). Hence, given the significance of synovitis in the initial phases of knee osteoarthritis, these individuals at risk of disease progression should become treatment targets, particularly through weight loss and exercise interventions as per the guidelines for knee osteoarthritis treatment (28–30). The monitoring and management of these patients can lead to a more efficient utilization of healthcare resources, particularly in light of the rising prevalence of the osteoarthritis. By utilizing patellofemoral grind test as a means of identifying individuals at risk of disease progression, clinicians can offer timely feedback to patients, encouraging them to engage in weight control and exercise interventions to enhance their overall health outcomes.

In addition to the aforementioned factors, our study also investigated the potential sex-specific differences in the association between patellofemoral grind and synovitis. In our analysis, we observed a stronger correlation between patellofemoral grind and synovitis in female patients. This finding is consistent with prior research indicating that women are at a higher risk of knee osteoarthritis and synovitis worsening compared to men (31, 32). It is postulated that this sex-specific difference may be attributed to hormonal factors, biomechanical differences, and differences in muscle strength (33). For instance, estrogen has been shown to have an impact on cartilage metabolism and inflammation, potentially increasing the vulnerability of women to knee osteoarthritis (34). Additionally, women tend to exhibit lower quadriceps strength compared to men, which may also contribute to the observed sex-specific differences (35). Especially in our observational cohort, women are all over 45 years old. Our findings underscore the importance of considering sex-specific differences in the assessment and management of knee osteoarthritis, particularly in relation to patellofemoral grind and synovitis.

The advantage of this study is that OAI provides a large number of samples to study the cross-sectional and longitudinal associations between patellofemoral grind and synovitis. In addition, a standardized scoring system greatly improves the reliability of the analysis. However, some limitations of this study should be acknowledged. First, the cross-sectional and longitudinal design of the study does not allow for the establishment of causality between patellofemoral grind and synovitis. Second, the reliance on self-reported data for the assessment of risk factors, such as knee injury history and surgery history, may introduce recall bias and affect the accuracy of our findings. Finally, although quadriceps weakness may lead to abnormal friction and stress in the patellofemoral joint, further mediation analysis is still needed to prove that the pathological state of patellofemoral plays a mediating role between the strength of quadriceps femoris muscle and the knee osteoarthritis phenotype.

## Conclusion

The findings of our study suggest a significant correlation between patellofemoral joint grinding and the progression and worsening of synovitis in knee osteoarthritis, both in a cross-sectional and longitudinal manner, particularly among female individuals. Therefore, it is imperative for healthcare professionals to closely monitor and manage these patients, while also implementing early weight loss and exercise interventions.

## Data availability statement

The datasets presented in this study can be found in online repositories. The names of the repository/repositories and accession number(s) can be found below: http://nda.nih.gov/oai/.

## Ethics statement

Ethical review and approval was not required for the study on human participants in accordance with the local legislation and institutional requirements. The patients/participants provided their written informed consent to participate in this study.

## Author contributions

HD, YW, ZF, WT, and JT contributed to the data acquisition. HD, ZF, and JT contributed to data analysis, interpretation, and drafted. HD, YW, WT, and JT conceived and designed the study. JT were in charge of the conceptualization, supervision and project administration. All authors contributed to the article and approved the submitted version.

## Funding

This research was funded by the Science and Technology Department of National Natural Science Foundation of China (No. 82260426), Medical Leading Discipline Orthopedics (Arthroscopy) Construction Project of Jiangxi Province, the Nanchang key Laboratory for Rehabilitation of Sports injuries (No. 2020-NCZDSY-009).

## Conflict of interest

The authors declare that the research was conducted in the absence of any commercial or financial relationships that could be construed as a potential conflict of interest.

## Publisher’s note

All claims expressed in this article are solely those of the authors and do not necessarily represent those of their affiliated organizations, or those of the publisher, the editors and the reviewers. Any product that may be evaluated in this article, or claim that may be made by its manufacturer, is not guaranteed or endorsed by the publisher.

## Footnotes

^1^ http://nda.nih.gov/oai/
